# Disseminated Histoplasmosis with Concurrent Hairy Cell Leukemia

**DOI:** 10.7759/cureus.6825

**Published:** 2020-01-31

**Authors:** Abdul Moiz Khan, Sheikh Raza Shahzad, Saleh Najjar, Llewellyn Foulke

**Affiliations:** 1 Internal Medicine, Albany Medical Center, Albany, USA; 2 Pathology, Albany Medical Center, Albany, USA

**Keywords:** hairy cell leukemia, histoplasmosis, immunosuppression, fungal infections

## Abstract

Histoplasmosis is an endemic fungal infection that can lead to disseminated disease, especially in immunosuppressed patients. Hairy cell leukemia is a rare, slow-growing hematological malignancy. Concurrence of histoplasmosis and hairy cell leukemia is extremely rare. We describe a 69-year-old male who presented with fever, dry cough, pancytopenia, multiple pulmonary nodules, and massive splenomegaly. Histoplasma urinary antigen was positive and disease was confirmed by biopsy of lung lesions. Peripheral smear showed ‘hairy cells’, and bone marrow biopsy revealed findings of hairy cell leukemia. The patient was treated with intravenous amphotericin, followed by oral itraconazole. After the initial treatment of infection, treatment for hairy cell leukemia was started with cladribine. We will discuss the principles of treating disseminated histoplasmosis in the setting of immunosuppression, and hairy cell leukemia with coexisting infection.

## Introduction

Histoplasmosis is the leading mycosis in terms of prevalence, hospitalization and mortality in the USA [1]. It is endemic in the regions of Ohio and Mississippi river valley in the USA. However, parts of Mexico, Central America and South America also have an increased incidence [2,3]. Transmission of histoplasmosis is classically associated with soil contaminated with bird or bat guano. Therefore, farming, and exposure to chicken coops, caves, old demolished buildings or areas where trees are cut down may lead to an increased risk of exposure [2]. Hairy cell leukemia is a rare hematological malignancy with an annual incidence of only around 1000 cases in the USA [4]. Infectious complications in hairy cell leukemia are common and potentially life-threatening. Most of the fungal infections are caused by Aspergillus and Candida in the setting of neutropenia from the leukemia itself, or myelosuppression from the chemotherapy [5]. However, to the best of our literature review, there are only two detailed case reports with concurrent hairy cell leukemia and disseminated histoplasmosis. Even after accounting for the cases reported in some large studies, the number is still estimated to be less than 10 [6, 7].

We present a case of disseminated histoplasmosis in a patient with previously undiagnosed hairy cell leukemia. We will discuss the principles of treating disseminated histoplasmosis in the setting of immunosuppression, and treating hairy cell leukemia with a coexisting active infection.

## Case presentation

A 69-year-old male with past medical history of hypertension presented with two weeks of worsening dyspnea, dry cough, and intermittent fevers up to 102 F (38.9 C). He also reported poor appetite, night sweats and mild left upper quadrant discomfort for two weeks. The patient had a small meat-packing plant in Upstate New York. In addition to the cattle meat, he also packaged game meat of moose, caribou and deer. He also kept cattle, horse, dogs, cats and chicken on his farm. Travel history was significant for a recent trip to Missouri a few months ago. He also had history of hiking trips and recreational visits to caves within the last one year. The patient was a life-long non-smoker, non-alcoholic with no illicit drug use. On physical exam, he had evidence of conjunctival pallor, scleral icterus, scattered rhonchi in bilateral lung fields and prominent splenomegaly.

**Figure 1:**
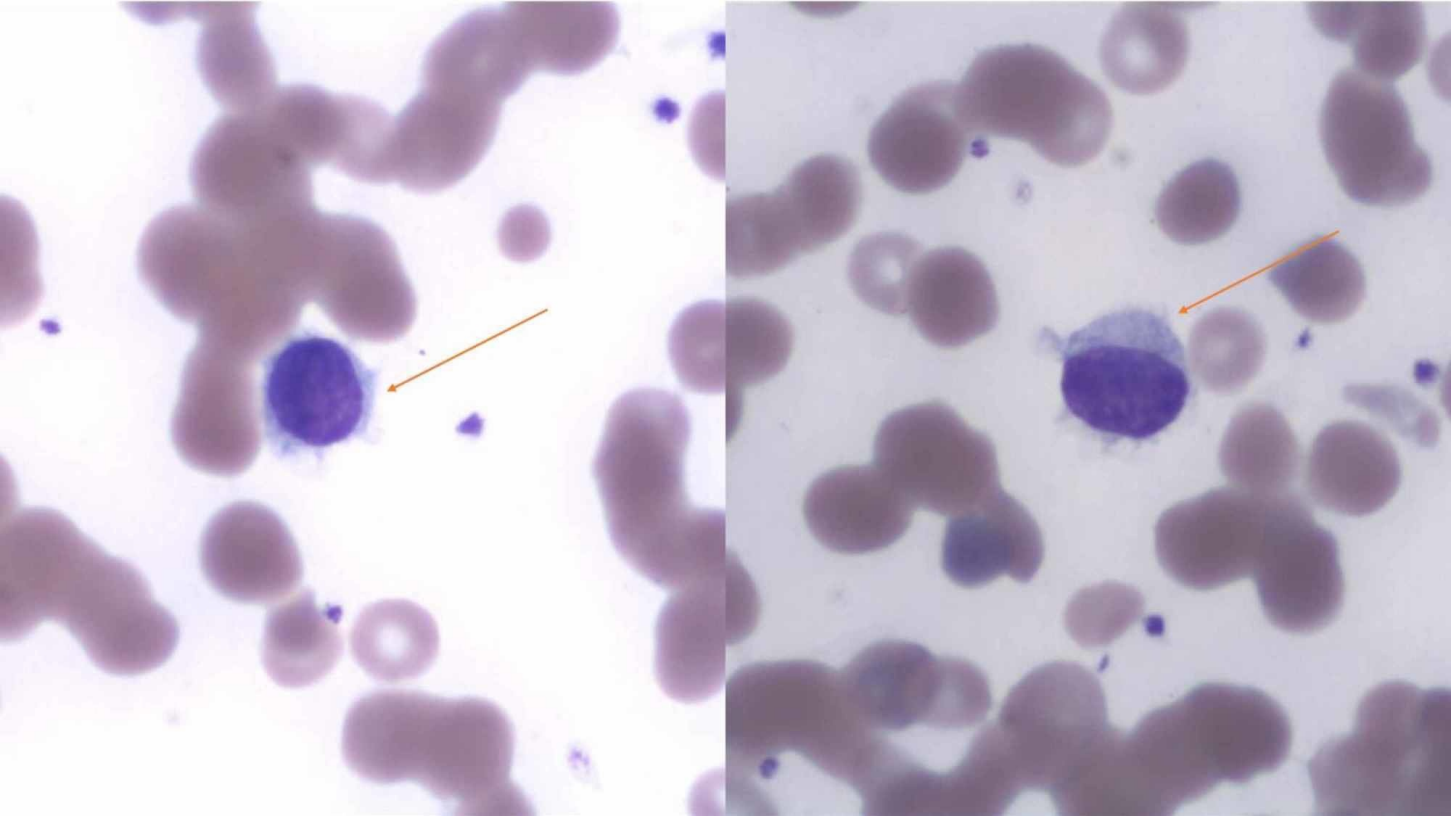
Peripheral smear showing leukocytes with spiculations and cytoplasmic projections consistent with “hairy cells” in both the figure panes.

**Figure 2:**
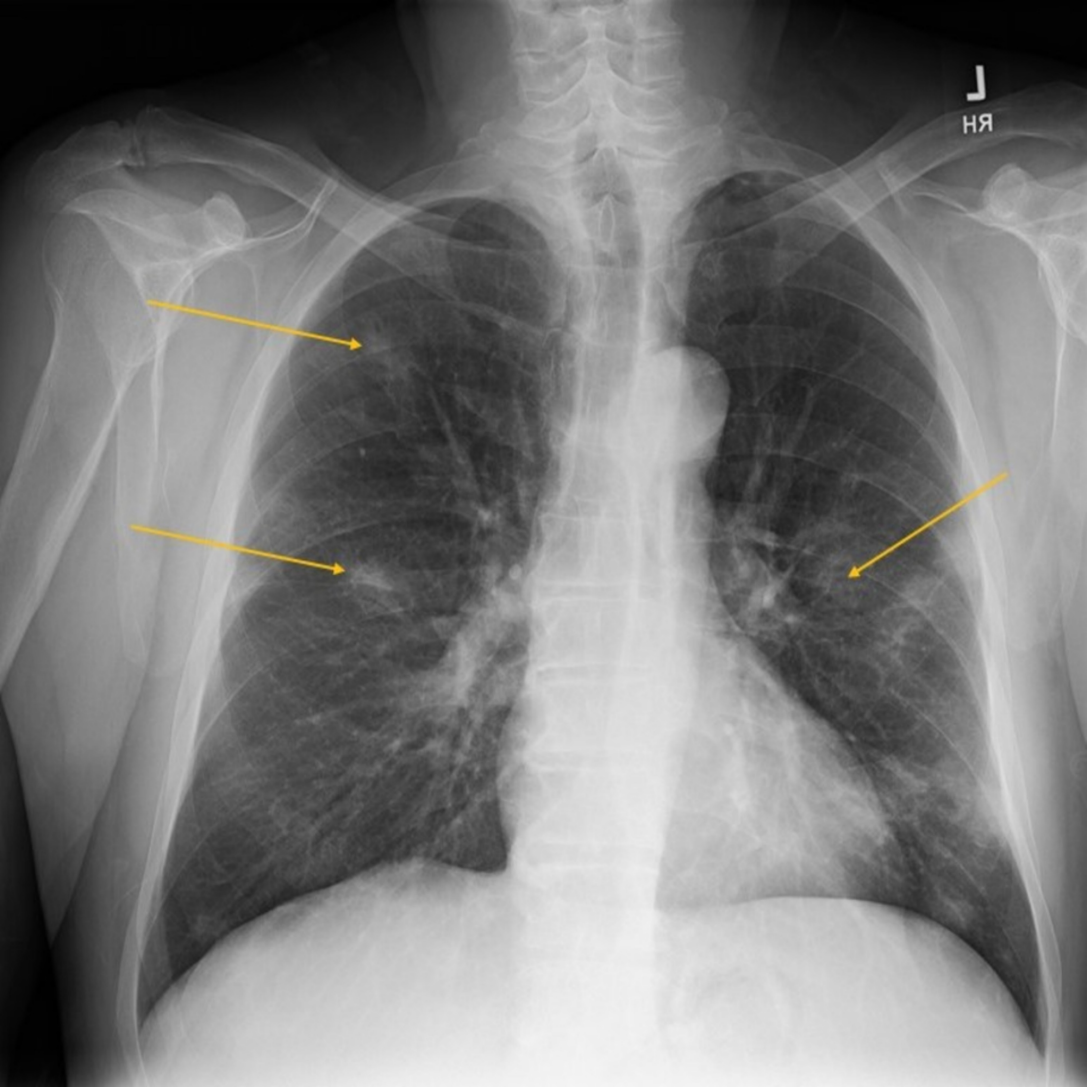
Chest X-ray showing multiple hazy opacities.

**Figure 3:**
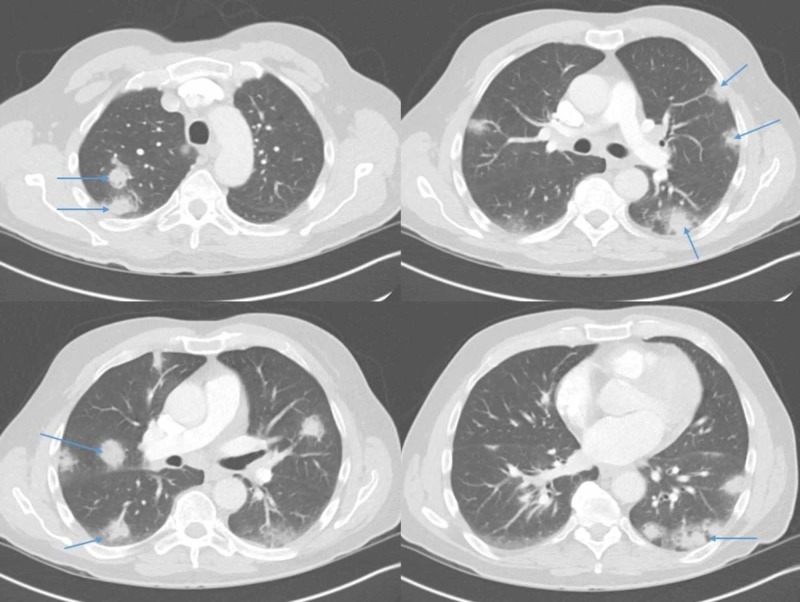
CT scan of the chest revealing numerous lung nodules seen in both lungs at multiple levels as demonstrated in the figure panes (see arrowheads).

**Figure 4:**
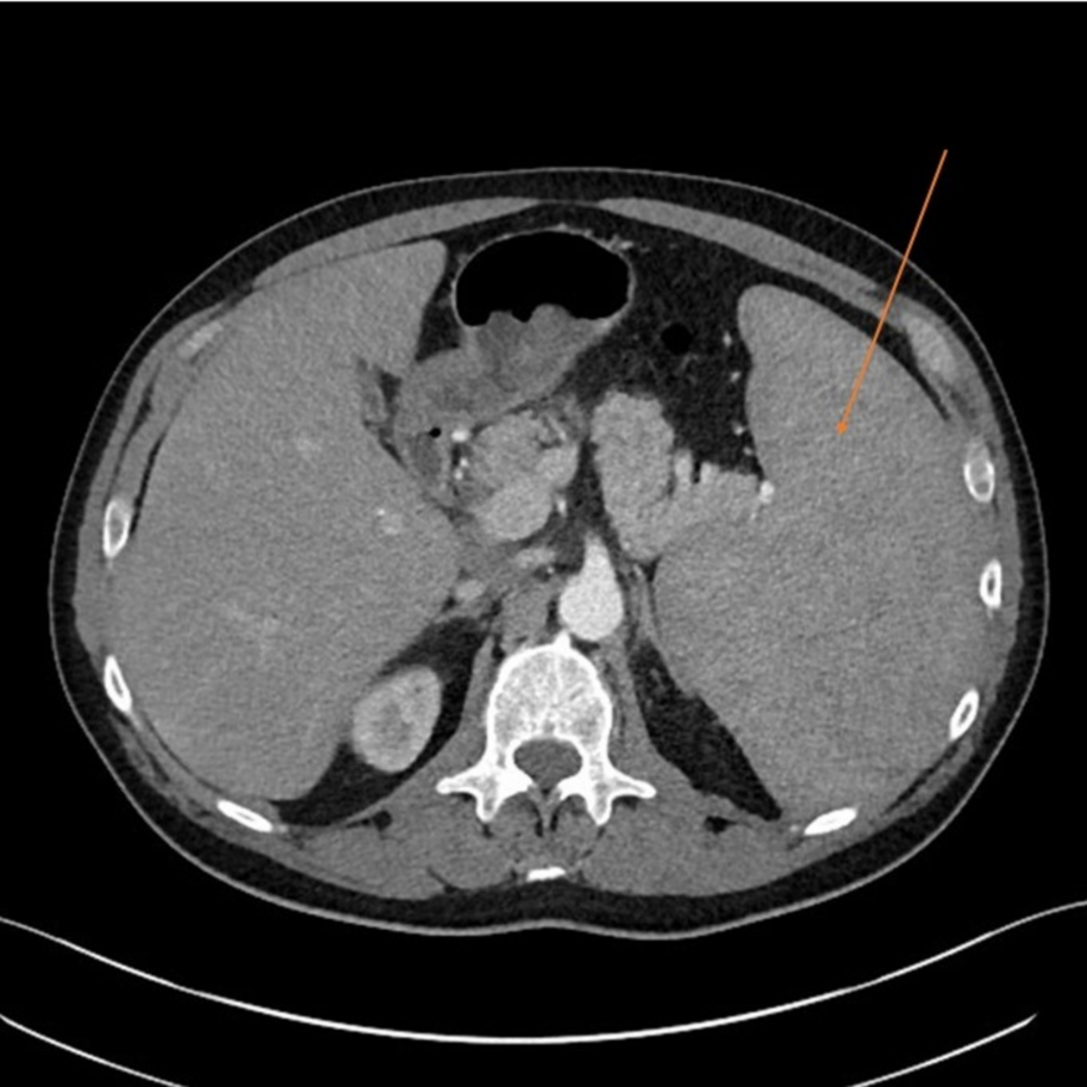
CT abdomen demonstrating severe splenomegaly, craniocaudal length of almost 20 cm.

Initial workup is as follows: Complete blood count (CBC) revealed pancytopenia with a white cell count of 2700/µl, absolute neutrophil count of 900/µl, hemoglobin (Hb) 7.4 g/dl, platelets 89,000/µl, mean corpuscular volume (MCV) 100; peripheral smear showed relative lymphocytosis, some leukocytes with spiculations and cytoplasmic projections consistent with “hairy cells” (Figure 1); raised inflammatory markers showed erythrocyte sedimentation rate (ESR) 120 mm/hr, C-reactive protein (CRP) 119 mg/L, ferritin 2492 ng/ml; elevated liver enzymes with hyperbilirubinemia showed alanine transaminase (ALT) 134, aspartate transaminase (AST) 129, alkaline phosphatase (ALK) 333 units/L, bilirubin 2.6 mg/dl; chest X-ray revealed numerous bilateral hazy opacities (Figure 2); CT chest revealed mild hilar lymphadenopathy, multiple nodular lesions at least 12 in each lung, up to 2.5 cm in diameter (Figure 3); CT abdomen and pelvis showed severe splenomegaly, craniocaudal dimension of 20 cm (Figure 4).

Based on patient’s presentation and preliminary workup, differential diagnosis included disseminated infections as well as hematological malignancies. We also considered that there might not be one unifying diagnosis per se, and patient could have an infectious process on top of an underlying malignancy that is causing the immunosuppression. Suspicion for autoimmune vasculitides was low, although they were also accounted for in the workup.

Based on the epidemiology, and frequent exposure to soil, animals and caves, testing for specific pathogens was done:

Histoplasma urinary antigen was positive at 6.75 ng/ml (Test measured levels between 0.4-19 ng/ml. Results <0.4 were reported as positive below limit of quantification, >19 were reported as positive above the limit of quantification).

Blastomyces urinary antigen and Cryptococcus serum antigen were negative. Francisella tularensis serology, Coxiella burnetii PCR, Galactomannan and Fungitell assay for Aspergillus, and testing for tick-borne illnesses (including Lyme, anaplasmosis, babesiosis) were negative.

Blood and urine culture for bacteria and fungi, human immunodeficiency virus (HIV) p24 antigen, quantiferon-TB and testing for hepatitis A, B and C was negative.

**Figure 5:**
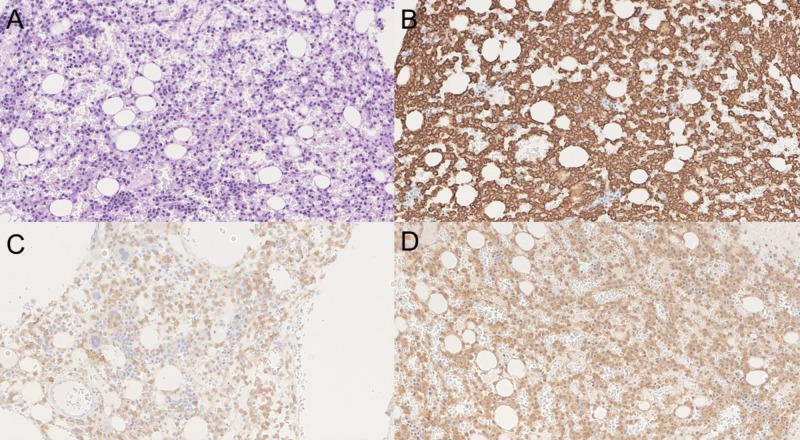
Bone marrow biopsy. Panel A is periodic acid–Schiff (PAS) stained image showing diffuse involvement of the bone marrow with loosely spaced medium-sized cells with oval indented nuclei and no distinct nucleoli; panel B shows that the cells are diffusely positive for CD20; panel C shows positivity for CD25; and panel D demonstrates that the cells are positive for mutation-specific BRAF V600E (BRAF valine to glutamic acid mutation) antibody. Overall, the morphology and staining pattern is consistent with hairy cell leukemia.

**Figure 6:**
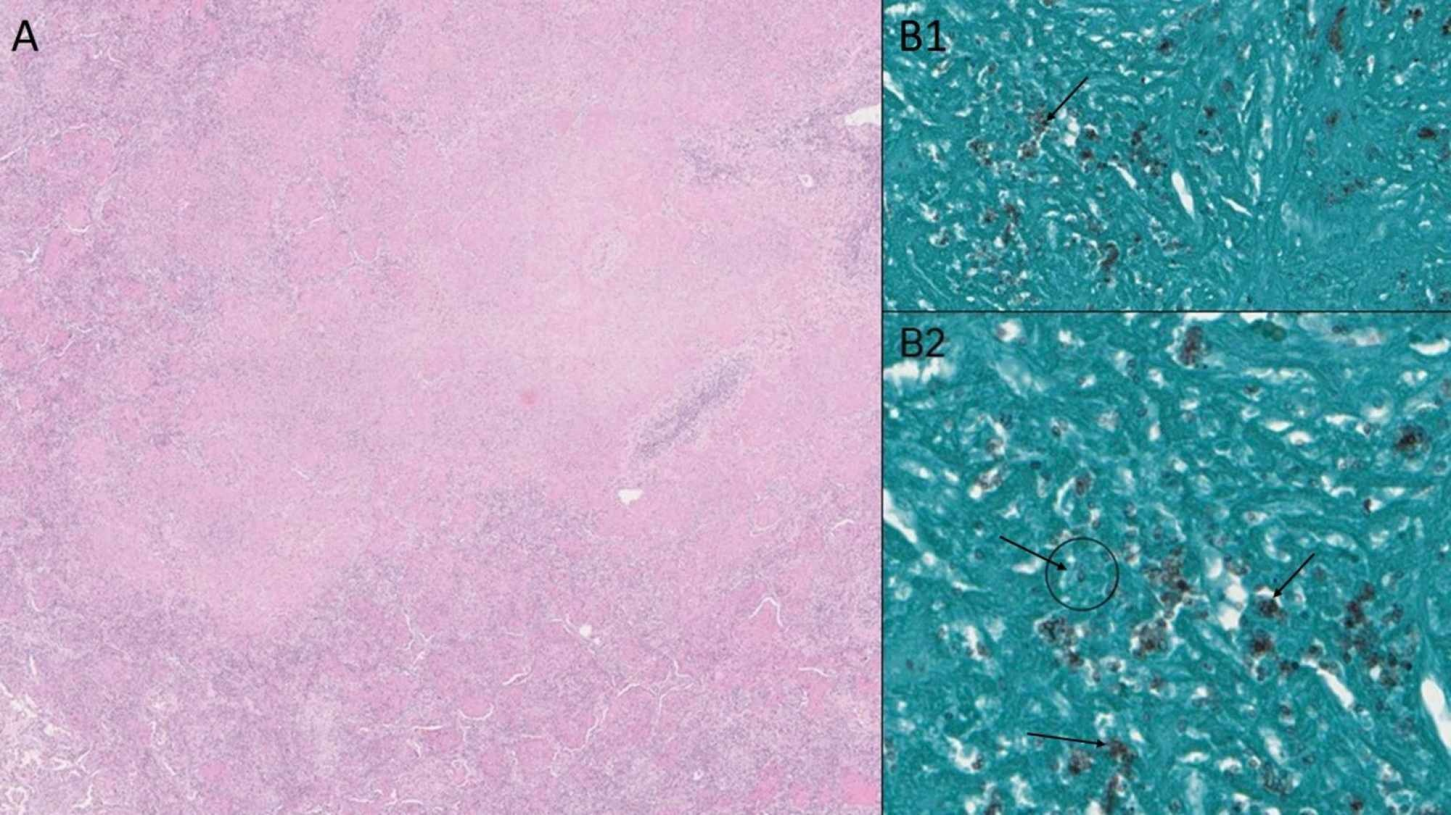
Lung biopsy. Panel A shows hematoxylin and eosin (H&E) stained lung lesion consisting of granulomatous reaction with caseation. Panel B1 and B2 reveal Gomori methenamine silver (GMS) stained aggregates of intracellular yeast-like organisms with narrow-based budding consistent with Histoplasma capsulatum (GMS stains fungal cell walls as black, see arrows).

Bone marrow biopsy demonstrated extensive CD20 positive B-cell lymphoma in a diffuse and interstitial pattern comprising approximately 50% of the marrow core. The neoplastic B-cells were positive for CD20, BRAF, CD25, CD123, CYCLIN D1 and Annexin A1. Reticulin stain demonstrated mild (1+) reticulin fibrosis. Bone marrow aspirate flow cytometric analysis revealed a kappa restricted B cell population (17%) positive for CD19, CD20, CD22, HLA-DR, FMC7, CD25, CD11c and CD103 and negative for CD10 and CD5. No expanded blast population was detected (see Figure 5). These findings along with the cells with cytoplasmic projections in the peripheral blood were consistent with “hairy cell leukemia”. Bronchoalveolar lavage (BAL) showed chronic inflammatory cells, and sparse bronchial epithelial cells. No viral cytopathic effect was present. Gomori Methenamine-Silver (GMS) demonstrated rare, equivocal, poorly preserved yeast-like forms. Since the BAL was indeterminate, video-assisted thoracoscopic wedge biopsy of the lung was done. It revealed nodular organizing pneumonia with extensive fibrinous exudates, increased histiocytes, chronic inflammation, and edema. Non-necrotizing granulomas with central suppurative necrosis and numerous fungal yeast forms consistent with Histoplasma were found (see Figure 6). c-ANCA, p-ANCA, atypical ANCA and ANA were negative.

The patient was empirically started on intravenous liposomal amphotericin after the initial CT findings and positive urinary histoplasma antigen. The diagnosis was confirmed by the lung biopsy and amphotericin was continued. Once the diagnosis of hairy cell leukemia was confirmed with bone marrow biopsy, it was decided to defer the treatment of leukemia given presence of active infection. After three weeks of treatment with amphotericin, the patient was transitioned to oral itraconazole and will be continued on it for at least one year. The patient had gradual clinical improvement. Histoplasma urinary antigen level decreased significantly (to <.5 ng/ml). Eight weeks after the initial presentation, he was started on cladribine for the treatment of hairy cell leukemia. He remained in close outpatient follow-up with both infectious disease and hematology. He showed great response to the treatment of both the Histoplasmosis and hairy cell leukemia with significant improvement in the symptoms over the next few months. At 14 months from the initial presentation, the patient has completed the antifungal treatment and the leukemia is in remission.

## Discussion

This case is an interesting example of epidemiology providing decisive clues about the diagnosis. From a clinical standpoint, features of histoplasmosis and hairy cell leukemia may overlap. Constitutional symptoms like fatigue, fever, night sweats, weight loss and anorexia, and clinical findings including splenomegaly, hepatomegaly, pancytopenia, lymphadenopathy, pulmonary infiltration and bony involvement can occur in both hairy cell leukemia and disseminated histoplasmosis [2,8]. In our patient, the clinical presentation had a cumulative effect of both the diseases.

Disseminated histoplasmosis in the setting of immunosuppression should be treated initially with intravenous liposomal amphotericin B for one to two weeks, followed by oral itraconazole for at least one year [9]. Liposomal formulation of amphotericin has shown better outcomes than the deoxycholate formulation [10]. If itraconazole is not tolerated, other antifungal agents like fluconazole, posaconazole, voriconazole or isavuconazole can be considered, although data on Isavuconazole is very limited [9,11]. Nevertheless, echinocandins (like micafungin) should never be used since they are ineffective against histoplasma [12].

One of the biggest challenges encountered in the treatment of hairy cell leukemia is the presence of an active infection. It is recommended to effectively treat the infection first before any myelosuppressive therapies like cladribine are instituted [13-15]. In some cases, interferon alpha can be used to improve the granulocyte count first, so as to enable the antibacterial or antifungal agents to be more effective [13,16]. Filgrastim may also have a beneficial role in the setting of severe neutropenia and active infection at diagnosis [17]. However, filgrastim showed no benefit in reducing the episodes of neutropenic fever in patients who did not have an infection at the time of initiation of treatment with cladribine [18]. Pentostatin has been used in a large study where the effect of infection at registration on the achievement of complete remission was ‘statistically’ not significant. Nevertheless, the complete remission rate was still lower in the infected patients (68%) as compared to the non-infected group (78%). Therefore, same strategy of infection control prior to the initiation of therapy is recommended [13,19]. Risk of infectious complications with newer agents like vemurafenib (BRAF serine‐threonine kinase inhibitor) is a subject that needs further investigation [14].

## Conclusions

Epidemiology and environmental exposures should serve as useful clues, especially in case of rare clinical presentations. In case of disseminated mycoses, attempt should be made to identify an underlying cause of immunosuppression, like malignancy, HIV/AIDS or medications like tumor necrosis factor alpha (TNF-α) inhibitors. Disseminated histoplasmosis in the setting of immunosuppression should be treated with intravenous liposomal amphotericin B for 1-2 weeks, followed by oral itraconazole for at least one year. Effective treatment of active infection should be ensured before initiation of myelosuppressive therapy for hairy cell leukemia.
